# Assessment of peripheral blood lymphocyte subsets in children with iron deficiency anemia

**DOI:** 10.1186/s12887-018-0990-5

**Published:** 2018-02-12

**Authors:** Sanaa S. Aly, Hanan M. Fayed, Ahlam M. Ismail, Gehan L. Abdel Hakeem

**Affiliations:** 1 Clinical and chemical pathology Department, Faculty of Medicine, South Valley University, Quena, Egypt; 2 Peditretic Department, Faculty of Medicine, South Valley University, Qena, Egypt; 3 Peditretic Department, Faculty of Medicine, Minia University, El Minya, 61511 Egypt

**Keywords:** Blood lymphocyte subsets, Children, Iron deficiency anemia

## Abstract

**Background:**

Iron plays an important role in body defense and essential for normal immune system development where its deficiency may result in an inadequate immune response. We aimed to assess the lymphocyte subsets in childhood iron deficiency anemia (IDA) with their laboratory correlations.

**Methods:**

Fifty IDA (< 18 years) and 25 age and sex-matched healthy children were enrolled and a complete history was obtained and clinical examination was performed. Complete blood count, serum iron, total iron binding capacity and serum ferritin, were performed. Flow cytometric determination of peripheral blood CD3+, CD4+, CD8+ T-lymphocytes and CD19+ B-lymphocytes and CD4/CD8 ratio were done.

**Results:**

Patients had significantly lower hemoglobin, Serum iron, ferritin levels and higher lymphocytic count in patients compared with controls (*p* = 0.001, 0.03, 0.001, 0.001 respectively). CD3 count and percentage were significantly lower in IDA patients compared to controls (*p* = 0.007 and 0.005 respectively).

There was a Significant reduction in the CD4 count, percentage and CD4/CD8 ratio in patients compared with controls (*p* = 0.001, 0.001 and 0.005 respectively) while there was no significant difference regarding CD8 count and percentage. No significant difference between the two studied groups regarding either CD19 count or percentage (*p* = 0.28 and 0.18 respectively) were found.

**Conclusions:**

IDA is associated with impaired cell-mediated immune response specifically T-cell mediated immunity.

## Background

Iron deficiency anemia is the most common preventable nutritional deficiency [1].

Iron and immunity are found to be in a close relationship. Iron utilization by invasive bacteria is controlled by genes/proteins which regulate iron flux to such organisms thus interfering with their growth. Moreover, through hepcidin and ferropotin, iron flux into the bacterial cell is controlled and this is mediated by the cells of innate immune system e.g. Monocytes, macrophages, and lymphocyte cells of the innate immune system. In addition, many effector molecules, e.g. haem oxygenase, toll-like receptors, hypoxia factor-1, NF-κB which are the regulatory molecules in the inflammatory response organize a multiplicity of cytokines, chemokines, reactive oxygen and nitrogen species at which either iron loading or depletion occur. This may adversely affect the ability of the cell to respond to the bacterial insult [2].

Iron has an essential role in surveillance of immune cells, particularly in lymphocytes, due to its growth-stimulating and differentiating properties [3]. Also, iron is necessary for monocyte/macrophage differentiation [4]. However, humeral immunity seems to be less affected by iron deficiency compared to cellular immunity [5].

Iron is also critical for enzymes involved in DNA synthesis, and the proliferative phase of lymphocyte activation is a Fe^−^demanding phase and it can be weakened during IDA [6] thus results in altered expression of cell markers that may contribute to the reduced T-cell proliferation [7].

However; there are three types of mature T cells based on their surface receptor expression; T helper cells (CD3+/CD4+), T-suppressor/cytotoxic cells (CD3+/CD8+) and γ/δ T cell (CD2+, CD3+, CD7+, CD4− and CD8−) that function as a type of cytotoxic cells [8].

Data on the effect of iron deficiency on immune function are confusing and contradictory. We aimed to assess the percentage and absolute counts of the peripheral blood lymphocyte subsets in children with iron deficiency anemia by a flow cytometric assay and correlate the results with the clinical and laboratory criteria of the affected children.

## Methods

### Patients

This multicenter, case-control study was conducted in Quena and El Minya cities in Egypt. It was carried out between January and October 2016. Patients were selected from the Outpatient Clinic at Quena (24 patients) and Minia (26 patients) University children’s hospitals.

Fifty patients with proven iron deficiency were enrolled and involved 23 males (46%) and 27 females (54%). Their age range was the 2–16 years.

#### Eligibility

Children aged 2–16 years diagnosed to have iron deficiency anemia based on laboratory investigations of hypochromic microcytic anemia and a history and examination correlating with anemia, low serum ferritin, low serum iron and increased total iron binding capacity. IDA patients fulfilled the following criteria: Hemoglobin < 10 g/dl, Mean corpuscular volume (MCV) < 80 fl, serum ferritin < 20 ng/ml.

#### Exclusion criteria

Patients with known or proved immune deficiency, patients with protein-energy malnutrition, patients with acute or chronic systemic illness known to alter the immune system, children with a history of receiving iron, other hematinic or multivitamins in preceding 3 months, chronic blood loss or recent acute blood loss, chronic illness, acute or chronic infection or parasitic infestation at the time of the study, history of taking immunosuppressant drugs, radiotherapy or chemotherapy were excluded from the study.

#### Controls

Twenty-five age and sex-matched apparently healthy children were selected from Pediatric growth clinic at Minia and Quena children’s University Hospitals as a control group. All patients and controls were examined carefully (general examination, anthropometric measures plotted on percentile growth charts, vital data as well as chest, heart, and abdominal examination).

Complete Blood Count (CBC) where neutrophils/lymphocyte and platelet/lymphocyte ratios were calculated, serum iron, serum ferritin, total iron binding capacity (TIBC), CD3, CD4, CD8 and CD19 counts and percentages also, the CD4/CD8 ratio was calculated.

The study was carried out in accordance with the World Medical Association’s Declaration of Helsinki and approved by the research ethics committee of Qena and Minia Universities. Informed written consents at the beginning of the study were obtained and all data were kept confidential and used for research purposes only.

### Blood sampling

Venous blood samples were collected from both patients and controls under complete aseptic conditions. Approximately 2 ml of blood was withdrawn in K3-EDTA anticoagulant tubes for complete blood counts, peripheral blood smears as well as flow cytometric analysis (immunophenotyping). About 3 ml of blood in a plain tube without any anticoagulant was left to clot and centrifuged at 3000 rpm (rpm) for 5 min. The serum was then separated and stored at − 70 °C till used for iron, serum ferritin, and TIBC.

Blood samples were withdrawn from IDA patients just prior to a scheduled iron therapy. The blood samples from the control group were taken while coming for follow-up of their growth. All controls had normal Hb levels for their age and sex with normal red blood cell indices. At the time of sampling, all patients and controls were apparently free from infection.

### Laboratory methodology

Complete blood count was performed using an automated blood counter (Sysmex KX-21N). Additionally, peripheral blood smears were stained with Leishman stain. The absolute neutrophil, lymphocyte and platelets counts were retrieved separately and used to calculate the neutrophil/lymphocyte ratio (NLR) and platelets lymphocyte ratio (PLR). Serum iron and TIBC were assayed using Cobas c311 automated chemistry analyzer (Roche Diagnostics, Germany). Serum ferritin was assayed by two-site sandwich direct immunoassay using Cobas e411 automated chemistry analyzer (Roche Diagnostics, Germany). The assay was performed according to the manufacturer’s instructions.

### Flow cytometric immunophenotypic analysis

T-Lymphocyte subsets in whole blood samples were enumerated using fluoro isothiocyanate (FITC) conjugated CD4 (Becton Dickinson, Bioscience, USA), phycoerythrin (PE) conjugated CD8 (Becton Dickinson, Bioscience, USA) and peridinium-chlorophyll-protein (Per-CP) conjugated CD3 (Becton Dickinson, Bioscience, USA). B-Lymphocyte subsets in whole blood samples were enumerated using phycoerythrin (PE) conjugated CD19 (Becton Dickinson, Bioscience, USA). Flow cytometric analysis was done by FACS Calibur flow cytometry with Cell Quest software (Becton Dickinson Biosciences, USA). An isotype-matched negative control was used with each sample. Forward and side scatter histogram was used to define the lymphocyte population (R1). The absolute counts and percentages of CD3, 4, 8 and 19 subsets of lymphocytes were calculated by multiplying the percentage (results of flow cytometer) by absolute lymphocyte counts (results of CBC).

## Statistical analysis

Data analysis was performed using statistical program for social science SPSS software version 16 (SPSS Inc., Chicago, IL, USA). Quantitative variables were presented as Mean ± standard deviation (SD). Qualitative variables were presented as number (No.) and percentage (%). The Chi-square was applied for qualitative variables. Data that were not normally distributed were expressed as median. Unpaired student t-test was used to compare between groups as regards quantitative variables while Mann-Whitney U test was used for non-parametric variables. Pearson correlation coefficient (r) was used to explore the relationship between quantitative variables. All statistical tests were two-tailed, and a probability (*p*-value) below 0.05 was considered statistically significant.

## Results

Seventy-five children were enrolled in this study, 50 patients with confirmed iron deficiency anemia (IDA) (their median age was 8 years). Twenty-three were males and 27 were females. Another healthy 25 age and sex-matched children were enrolled as controls (12 were males and 13 were females and the median age was 7.2 years (Table 1).

**Table 1:** Some demographic data of patients and control group

Median	patients	controls	*p*-value
Age (year)	8	7.2	0.41
Gender No (%)			
Male	23 (46%)	12 (48%)	0.35
Female	27 (54%)	13 (52%)	
Organomegaly (%)	10%	0%-	–
Weight (Kg) centile (th)	25	50	0.001*
Height centile (th)	10	25	0.001*
BMI (kg/cm2) centile (th)	30	35	0.04*

*Significant (*p* < 0.05)

Patients had significantly lower hemoglobin concentration, Serum iron (*p* = 0.001 for all), ferritin and higher lymphocytic count (*p* = 0.03) in IDA patients compared to the controls. There was no significant difference between the two groups regarding white blood cell count (WBC), neutrophil count, neutrophils/lymphocyte ratio and platelets count (Table 2).

**Table 2:** Some laboratory finding of patients and controls

Parameter (Median)	Patients	Control	*p-*value
Hb level (g/dl)	9.7	11.5	0.001*
WBC count (× 10^9^/L)	7.300	6.200	0.118
Neutrophil count (×10^9^/L)	2659	2666.00	0.83
Lymphocyte count (×10^9^/L)	3386	2632.00	0.03*
Platelet count (×10^9^/L)	375.000	334.000	0.19
Neutrophil Lymphocyte ratio	0.78	1.04	0.11
Platelets lymphocyte ratio	0.9	1.1	0.09
Serum iron (μg/dl)	70	82	0.001*
Serum ferritin (μg/dl)	18	34	0.001*
TIBC (μmol/L)	423	303	0.001*

*Hb* hemoglobin, *WBC* white blood cell count, *TIBC* total iron binding capacity

* Significant (*p* < 0.05)

CD3 count and percentage were significantly lower in IDA patients compared to controls (*p* = 0.007 and 0.005 respectively).

IDA patients showed a significant reduction in the count and the percentage of CD4 compared with controls (*p* = 0.001 for both). No significant difference between both study groups regarding CD8 count and percentage (*p* = 0.22 and 0.71 respectively). The CD4/CD8 ratio was significantly lower in IDA patients (*p* = 0.005). No significant difference between the two studied groups regarding either CD19 count or percentage (*p* = 0.28 and 0.18 respectively) (Table 3).

**Table 3:** Some immunological parameters for patients and controls

Parameter	Patients	Control	*p-*value
Median CD3 count	328	523.00	0.007*
Median CD3%	12.94	18.72	0.005*
Median CD4 count	213	397.00	0.001*
Median CD4%	7.63	14.34	0.001*
Median CD8 count	69	96.00	0.22
Median CD8%	2.80	3.06	0.71
Median CD4/CD8 ratio	1.72	2.71	0.005*
Median CD19 count	91	124.50	0.28
Median CD19%	3.37	4.13	0.18

* Significant (*p* < 0.05)

Significant positive correlation was found between serum iron level and absolute neutrophil count (*p* = 0.02, *r* = 0.27), CD4% (*p* = 0.001, *r* = 0.5) and CD4/CD8 ratio (*p* = 0.002, *r* = 0.35) while significant negative correlation was found between serum iron level and CD19 count (*p* = 0.02, *r* = 0.27) (Table 4).

**Table 4:** Correlation between serum iron and some immunological markers

	ANC		ALC	CD3 Count	CD3 %	CD4 Count	CD4 %	CD8 Count	CD4 %	CD4/CD8 ratio	CD19 Count	CD19 %
Serum iron (mg/dl)	*p*	0.02*	0.08	0.5	0.14	0.5	0.001*	0.05	0.19	0.002*	0.02*	0.69
	*r*	0.27	−0.2	−0.07	0.17	0.08	0.5	−0.22	−0.14	0.35	−0.27	−0.05

*Significant (*p* < 0.05); *ANC* absolute neutrophil count, *ALC* absolute lymphocyte count. *r* = correlation coefficient

## Discussion

Cellular and humoral immunities are mediated by lymphocytes (T and B) respectively. T lymphocytes are formed by the thymus gland and are released into the peripheral blood constituting more than 60% of the whole lymphocytic count [8]. Two types of mature T lymphocytes are formed. The T helper cells which express CD3+/CD4+ cell markers and T suppressor/cytotoxic cells which express CD3+/CD8+ cell markers. Mature B lymphocytes, however, are formed in bone marrow and express CD19 cell marker.

Iron was confirmed to be a vital element for immune system development and play an important role in the integrity of the immune system [9–11]. In the present study, IDA patients had a significant decrease in the total lymphocyte count compared to controls. Regarding lymphocytes subpopulation, T-lymphocytes (patients had a significantly lower number and percentage of CD3 positive cells) were significantly decreased while B lymphocytes (presented by CD19 positive cells) showed no significant difference compared with healthy children. This comes in agreement with the results of Kuvibidila et al., [12] who confirmed that T lymphocytes are much affected with iron than B lymphocytes.

Regarding T-lymphocyte subsets, the percentage and the absolute count of T helper cells (CD4 positive) were significantly lower compared to controls. Omara and Blakley, [13] proved a lower delayed hypersensitivity response (where CD4+ lymphocytes are the key marker) and decreased lymph proliferative activities in mice fed an iron-deficient diet than those fed a normal or supplemented iron diet. These results suggested impaired T-helper cell response in iron deficiency anemia [14].

The T-lymphocyte inhibition mechanisms in IDA patients are not fully understood. CD4+ lymphocytes are affected by iron deficiency which prevents the development of immune response against different pathologic challenges [15]. The function of different enzymes involved in cell-cycle control, such as the ribonucleotide reductase (involved in DNA synthesis during cell cycle S phase) is depressed by Intracellular iron deprivation [16, 17]. Iron restriction can also inhibit phosphatidylinositol-4, 5-biphosphate hydrolysis, and kinase C protein activity, both of which are important steps in the intracellular signaling cascade which is initiated by T cell activation [12]. Saldanha-Araujo and Souza, [18] and Markel et al., [19] reported that T lymphocytes iron uptake and their cellular proliferation is altered in IDA. Furthermore, the proliferation of T helper lymphocytes as Th-1 is much sensitive to the changes in the iron levels compared to the Th-2 lymphocyte. This is attributed to the difference in transferrin-related iron uptake [19].

Our results showed insignificant alteration in the percentage and the absolute count of CD8 positive lymphocytes. This is in agreement with Das et al., [20] and Mullick et al., [21, 22]. While Thibault et al., [23] found a decreased in the IL-2 production in IDA patients which was explained by alteration in CD8 positive lymphocyte maturation.

Iron was found to be a vital component of peroxide and nitrous oxide–generating enzymes. These enzymes are required for the proper immune cell function. Also, iron is involved in the regulation of cytokine production through its influence on second-messenger systems [24].

Phytohemagglutinin-induced lymphocyte proliferation and delayed-type hypersensitivity responses were found to be reduced in nutritional iron deficiency with relative preservation of humoral immunity [17]. Moreover, iron deficiency in humans can alter the cytokine expression profile of the activated lymphocytes [16].

The altered levels of some interleukins (IL) and cytokines (e.g. IL-2, IL-1, IL-6, TNF-α, IL-4, IL-12p40, IFN-γ, and IL-10) might result in immune system impairment in IDA patients [25, 26]. Also, altered cell marker expression may contribute to reduced T cell proliferation during iron deficiency [12].

Galan and colleagues, [27] reported a reduction in interleukin-2 production by activated lymphocytes in iron-deficient subjects. The release of interleukin-2 is required for communication between lymphocyte subsets and natural killer cells but it does not appear to be the only cytokine that is altered by iron status [15].

Regarding anti-microbial host responses, Iron plays important roles first by synergistic anti-microbial radical formation action [28–30] and second, by direct alteration of both immune cell proliferation and anti-microbial immune effector pathways [31]. Thus, the host immune system affects the availability of iron for microbes via the activity of cytokines.

In our study, the CD4: CD8 ratio was lower in children with IDA. This was in agreement with Das et al., [20] and Mullick et al., [21, 26]. Iron deficiency alters the proportion and function of various T cell subsets. Occasionally, the total lymphocyte count in the peripheral blood is low. All these changes are reversed by iron therapy. An altered immune response after iron administration may indicate the existence of an unsuspected functional iron deficiency. Several previous studies confirmed that iron supplementation improved the different subsets of the mature T-cell count and this is agreeing with the results of Sejas et al., [32] who confirmed that iron supplementation in children can significantly accelerate proliferation and maturation of the circulating immature lymphocyte subpopulations [33].

Some limitations had faced our study. Due to cultural and traditional issues, a lot of patients refused to be included in the study which led to a small sample size of the study.

## Conclusions

Iron deficiency anemia (IDA) in children can alter the lymphocyte subset in the absence of other factors challenging the immune system. Studies involving a larger number of children, belonging to well-defined age groups from different geographic areas are recommended for conclusive interpretation in the future.

## Abbreviations

CBC: Complete Blood Count
CD: Cluster differentiation
IDA: Iron deficiency anemia
IFN-γ: Interferon gamma
IL: Interleukin 1
Th1: T-helper 1
TIBC: Total iron binding capacity
TNF: Tumor necrosis factor
